# Dysregulation of epithelial differentiation architecture underlies field cancerization in head and neck cancer

**DOI:** 10.64898/2026.10.12.757511

**Published:** 2026-10-14

**Authors:** Claire Mills, Yin Huang, Chan Mi Lee, Connor Finkbeiner, Rachel K. Lex, Dominik J. Otto, Ruijin Yang, Hoku West-Foyle, Daniel Jones, Annalyssa Long, Sam Minot, Emma Nguyen, Pushpa Itagi, Samantha Schuster, Anna Elz, Lena Schroeder, Neal Futran, Emily Marchiano, Rocco Ferrandino, Rosa Ana Risques, Gavin Ha, Anshu Bandhlish, Brittany Barber, Manu Setty, Slobodan Beronja

## Abstract

Head and neck squamous cell carcinoma (HNSCC) has exceptionally high recurrence rates, exceeding 50%, contributing to mortality and morbidity. This recurrence rate is proposed to be due to field cancerization, a phenomenon where tumor-adjacent “field” tissue that appears histologically normal harbors molecular changes that prime it for subsequent tumor growth. Current models attribute field cancerization to shared driver mutations between tumors and adjacent at-risk tissue, but this assumption has never been directly tested. To comprehensively characterize this phenomenon, we profiled patient-matched tumor, normal-adjacent field (NAF), contralateral normal, and blood samples from HPV-negative HNSCC patients using whole genome sequencing, targeted driver gene sequencing, single-nucleus multiome profiling, spatial transcriptomics and functional proliferation assays. While histologically normal NAF tissues did harbor expanded mutant clones, these mutations rarely overlapped with paired tumors and were not shared across patients, suggesting molecular features beyond mutations are altered in NAF. Single-cell multiomics revealed that NAF epithelium loses its normal organizational hierarchy: basal stem cell markers persist in differentiated cells, while terminal differentiation programs fail to activate, with reconfiguration of both gene regulatory networks and cell-cell interactions. Proliferating cells appeared in tissue layers normally restricted to post-mitotic cells, a pattern typically seen only in overt dysplasia. Within this disrupted hierarchy, a distinct epithelial state arose in NAF that expressed both normal epithelial and tumor-associated markers, which we localized to the parabasal layers using immunofluorescence and spatial transcriptomics. Together, our findings demonstrate that field cancerization operates through epithelial hierarchy disorganization that disrupts differentiation, generates cells with tumor-like features, and may create a permissive environment for the expansion of cancer-associated mutations, all within tissue that appears histologically normal.

